# Bi‐directional Relationship between Traumatic Brain Injury and Dementia in Older Veterans

**DOI:** 10.1002/alz70860_100378

**Published:** 2025-12-23

**Authors:** Carrie B. Peltz, Jennifer S Albrecht, Amber L Bahorik, Feng Xia, Raquel C. Gardner, Kristine Yaffe

**Affiliations:** ^1^ NCIRE‐The Veterans Health Research Institute, San Francisco, CA, USA; ^2^ San Francisco Veterans Affairs Health Care System, San Francisco, CA, USA; ^3^ University of Maryland, School of Medicine, Baltimore, MD, USA; ^4^ University of California, San Francisco, San Francisco, CA, USA; ^5^ Sheba Medical Center, Joseph Sagol Neuroscience Center, Ramat Gan, Israel; ^6^ Department of Psychiatry, Neurology, and Epidemiology and Biostatistics University of California San Francisco School of Medicine, San Francisco, CA, USA

## Abstract

**Background:**

The risk of both traumatic brain injury (TBI) and dementia increase with older age. TBI is associated with increased risk of dementia. However, few studies have examined whether the relationship between TBI and dementia may be bi‐directional. The objective of this study is to examine incidence rates of dementia in older adults before and after an acute TBI.

**Method:**

We identified Veterans aged ≥55 with acute TBI (concurrent International Classification of Disease (ICD) TBI code + Emergency Department visit + brain imaging) using Veterans Health Administration data from 10/1/1999‐9/30/2021. We matched 3:1 to a non‐TBI comparison group on age, sex, race/ethnicity, and visit date (TBI visit date for TBI group vs. random visit date for comparison group). Dementia was identified from ICD codes one‐year pre/post‐TBI in the TBI cohort and over a two‐year period in the non‐TBI cohort. All Veterans with prevalent dementia one year before the study period were excluded. Incident dementia was calculated per 1000 person‐years with 95% confidence intervals (CI). Incidence rate ratios (IRR) and 95% CI were calculated comparing pre‐TBI to post‐TBI and to the non‐TBI comparison group.

**Result:**

We included 13,801 Veterans with acute TBI and a properly balanced comparison group of 41,403 Veterans without TBI (average age 77.8 years, 96.5% male). The acute TBI group had a much higher incidence of dementia pre‐TBI compared to background rates in the non‐TBI comparison group [58.1 per 1000 person‐years (95%CI 53.7‐62.5) vs. 18.5 (95%CI 17.5‐19.5)] for an IRR of 3.1 [(95%CI 2.9‐3.4), *p* <0.001]. As expected, Veterans with TBI had higher dementia incidence rates in the year post‐TBI [72.3 per 1000 person‐years (95%CI 67.0‐77.6)] compared to the year pre‐TBI with an IRR of 1.24 [(95% CI 1.12‐1.38, *p* <0.001)].

**Conclusion:**

The relationship between TBI and dementia is bi‐directional, with three times higher incidence rates prior to TBI compared to Veterans without TBI, and even higher rates post‐TBI. Older adults with dementia should be referred for TBI prevention and monitored should a TBI occur.

